# Incidence of Hyponatremia During a Continuous 246-km Ultramarathon Running Race

**DOI:** 10.3389/fnut.2019.00161

**Published:** 2019-10-11

**Authors:** Adam D. Seal, Costas A. Anastasiou, Katerina P. Skenderi, Marcos Echegaray, Nikos Yiannakouris, Yiannis E. Tsekouras, Antonia L. Matalas, Mary Yannakoulia, Fani Pechlivani, Stavros A. Kavouras

**Affiliations:** ^1^Hydration Science Lab, Arizona State University, Phoenix, AZ, United States; ^2^Department of Nutrition and Dietetics, Harokopio University, Athens, Greece; ^3^Department of Biology, University of Puerto Rico at Cayey, Cayey, Puerto Rico; ^4^Technological Educational Institution of Athens, Athens, Greece

**Keywords:** dehydration, fluid balance, electrolyte balance, hypohydration, sweat, sodium, heat, thermoregulation

## Abstract

**Purpose:** The purpose of this observational study was to examine the incidence of exercise-associated hyponatremia (EAH) in a 246-km continuous ultra-marathon.

**Methods:** Over 2 years, 63 male finishers of the annual Spartathlon ultra-marathon foot race from Athens to Sparta, Greece were included in the data analysis. A blood sample was drawn from an antecubital vein the day before the race as well as within 15 min post-race and analyzed for sodium concentration. During the second year of data collection, blood was also drawn at the 93-km checkpoint (*n* = 29). Height and weight were measured pre and post-race.

**Results:** Mean race time of all subjects was 33 ± 3 h with a range of 23.5 and 36.0 h. Of the 63 finishers recruited, nine began the race with values indicative of mild hyponatremia. Seven runners were classified as hyponatremic at the 93-km checkpoint, three of whom had sodium levels of severe hyponatremia. After the race, 41 total finishers (65%) developed either mild (*n* = 27, 43%) or severe hyponatremia (*n* = 14, 22%). Mean change in bodyweight percentage and serum sodium from pre-race to post-race was −3.6 ± 2.7% (−2.5 ± 1.9 kg) and −6.6 ± 5.6 mmol·L^−1^, respectively. Pre-race serum sodium level was not a significant predictor of post-race serum sodium levels (β = 0.08, *R*^2^ = 0.07, *P* = 0.698), however, there was a significant negative association between change in bodyweight percentage and post-race serum sodium concentration (β = −0.79, *R*^2^ = 0.29, *P* = 0.011).

**Conclusion:** The incidence of EAH of 52 and 65%, when excluding or including these individuals with pre-race hyponatremia, was the highest reported in current literature.

## Introduction

With the increasing popularity of ultra-marathon foot races (more than 42-km), recent studies have begun to examine the concomitant pervasiveness of exercise-associated hyponatremia (EAH) in these events (1–5). This condition is defined as a serum sodium concentration <135 mmol·L^−1^ during or after 24 h of exercise. EAH usually occurs as result of excessive hypotonic fluid intake coupled with high rates of sweat sodium excretion; however, other potential risk factors have been identified including the composition of ingested fluid, race time, body mass index, and the non-osmotic stimulation of the hormone vasopressin (1, 6). The decrease in plasma sodium causes a shift of fluid into the intracellular space resulting in possible cell swelling and the associated symptoms of nausea, vomiting, confusion, lung congestion, and increased intracranial pressure (7). Under normal conditions, serum sodium should be corrected by the renal system; however, during exercise increase in renal sympathetic nerve activity and activation of the renin-angiotensin system reduce urine flow. This reduction in urine impedes the ability of the renal system to excrete hypotonic fluids and correct the decrease of plasma sodium (7).

Varying results have been reported regarding the Incidence of EAH in ultra-endurance events. In 2015 and 2016, Chlibkova et al. reported ~11.5% of competitors in ultra-endurance events developed EAH. Both of these studies yielded positive correlations between body mass loss and plasma sodium (4, 5). However, in a 2014 study by the same group, the authors reported a Incidence rate of only 5.7% (3). In 2015, Cairns and Hew-Butler investigated the Great North Walk 100-mile ultra-marathon and reported an EAH rate of 27% (8). Conversely, Lebus et al. reported over 50% of competitors in the 161-km Rio Del Lago endurance run developed EAH, representing the highest Incidence recorded at a single event in current literature (9). In this study, there was no significant association between change in body mass and post-race serum sodium.

Numerous data exist outlining increased cardiovascular strain and performance decrements seen with >2% dehydration in warm/hot environments, even in the absence of thirst perception (10–12). Additionally, a recent study using real-time observations during a 120-km ultra-marathon showed runners failed to match fluid intake to fluid loss resulting in an average of 3.6 ± 2.3% dehydration (13). As mentioned earlier, this could affect performance in endurance events, thus indicating competitors should prevent dehydration more than 2% of body mass. However, excessive consumption of hypotonic fluids at a rate greater than fluid loss is a risk factor for development of EAH. A 2002 study published in the *New England Journal of Medicine* examined 488 runners in the Boston marathon. The researchers reported the risk of mild (<135 mmol·L^−1^) and severe (<130 mmol·L^−1^) hyponatremia was 30 and 70% greater in runners who gained 3–4.9 kg during the 42-km race (1). Therefore, careful consideration should be given to match fluid intake to sweat loss without over-drinking during ultra-marathons, especially for those runners with high sweat sodium content. In such circumstances, it has been proposed that sodium supplementation may help maintain sodium levels, thereby attenuating the risk of EAH (14–16). Vrijens and Rehrer examined plasma sodium concentrations in individuals performing a 3 h cycling protocol at low intensity. After 3 h, participants ingesting a beverage containing sodium had higher plasma sodium concentrations as compared to a water only group (17). Additionally, Koenders et al. found that participants following a diet with higher sodium before an endurance exercise event maintained plasma sodium concentrations more effectively as compared to those following a lower sodium diet (18).

Although most studies support the intake of sodium as a necessary measure during prolonged exercise, some recent data suggest sodium supplementation is not necessary during ultra-endurance events due to innate homeostatic mechanisms (19). However, these data were collected in cooler environments not conducive to excessive sweat rates. Hoffman and Stuemplfle examined the effects of sodium supplementation in athletes competing in a 161-km foot race with temperatures reaching 39°C. In this study, data revealed a weak positive correlation between post-race serum sodium and supplemental sodium intake; however, only 6–8% of the variability in post-race serum sodium was explained by sodium intake (20).

Contrasting data such as these may highlight the difference in individual needs throughout longer extreme endurance events. Sodium concentration in sweat can vary greatly between individuals (20–80 mmol·L^−1^) due to genetic factors and heat acclimation, with acclimated individuals losing less sodium (14, 21, 22). Considering variability in sweat sodium concentration, individualized sodium intake before and during extreme ultra-marathons may be pertinent to the reduction of EAH. McDermott et al. in the National Athletic Trainer's Association position statement, indicate that participation in events under intense environments may necessitate sodium supplementation, especially in individuals with sweat sodium concentrations >60 mEq·L^−1^. Therefore, the authors conclude assessment of sweat-electrolyte concentration should be performed before an individualized sodium supplementation plan is implemented (23).

Although numerous data exist regarding the Incidence of EAH in more popular shorter races, to the author's knowledge few studies have examined the Incidence of EAH in extreme ultra-marathons. Therefore, the purpose of this observational study was to examine the Incidence of EAH in a 246-km continuous ultra-marathon. It was hypothesized that due to the tremendous length and wide range of ambient conditions encountered in this particular ultra-marathon, the Incidence rate of hyponatremia would be high as compared to previous literature.

## Materials and Methods

Over the span of 2 years, we recruited 164 runners of the 246-km Spartathlon ultra-marathon foot race (http://www.spartathlon.gr/en). From the 164 originally recruited, only 63 runners (males, age: 41.9 ± 7.8 y, height: 1.74 ± 0.07 m, weight: 68.1 ± 6.6 kg) that completed the race (38% completion rate) were included in the data analysis. This race takes place annually in Greece during the last week of September. It begins every year from Athens on the last Friday of September at 7:00 am and finishes in Sparta by Saturday 7:00 pm. The Spartathlon is a point-to-point race with elevation ranging from sea level to 1,200 meters over tarmac road, trail, and mountain footpath. Around the midpoint point of the race lays a segment where runners climb 960 m over a span of 13-km. During the race, athletes must cover the first 81-, 124-, 148-, 172-, and 195-km of the race within 9:30, 15:30, 19:30, 23:30, and 27:00 h, respectively, to avoid being forced out of the race. To be considered a finisher, the 246-km course must be covered within 36 h. Ambient temperatures in this region ranged between 5°C at night and 36°C during the day. High carbohydrate food including bread, cookies, fruits, cereal bars, rice, yogurt, and pasta along with various beverages were consumed *ad libitum* from checkpoints at 75 locations throughout the 246-km course.

All procedures performed in studies involving human participants were in accordance with the ethical standards of the institutional and/or national research committee and with the 1964 Helsinki declaration and its later amendments or comparable ethical standards. Written informed consent was obtained from all volunteers prior to their participation in the study.

Height and weight were measured before the race in shorts and t-shirts without shoes using a stadiometer accurate to 0.5 cm and digital body mass scale accurate to 100 g. Body mass was also measured within 15 min post-race. A 10-mL blood sample was drawn from an antecubital vein the day before the race as well as within 15 min post-race. During the second year of data collection, blood was also drawn at the 93-km checkpoint (*n* = 29). Serum sodium was measured by selective electrode conductivity in an automated analyzer (Ektachem DT60 II system; Eastman Kodak Co, Rochester, NY). Biochemical EAH was defined as serum sodium levels <135 mmol·L^−1^.

Results are presented as means ± standard deviation. Shapiro-Wilk test was used to ensure normality before analysis. Paired *t*-tests were used to compare pre and post-race values and simple regression analysis was used to define the relationship between percent change in body mass and pre and post-race serum sodium levels as well as pre and post-race serum sodium. For all statistical tests, significance was set at *P* < 0.05. All statistical analysis was performed using JMP Pro 13 statistical software (version 13.0, SAS Inc., Cary, NC, USA).

## Results

**Figure 1:**
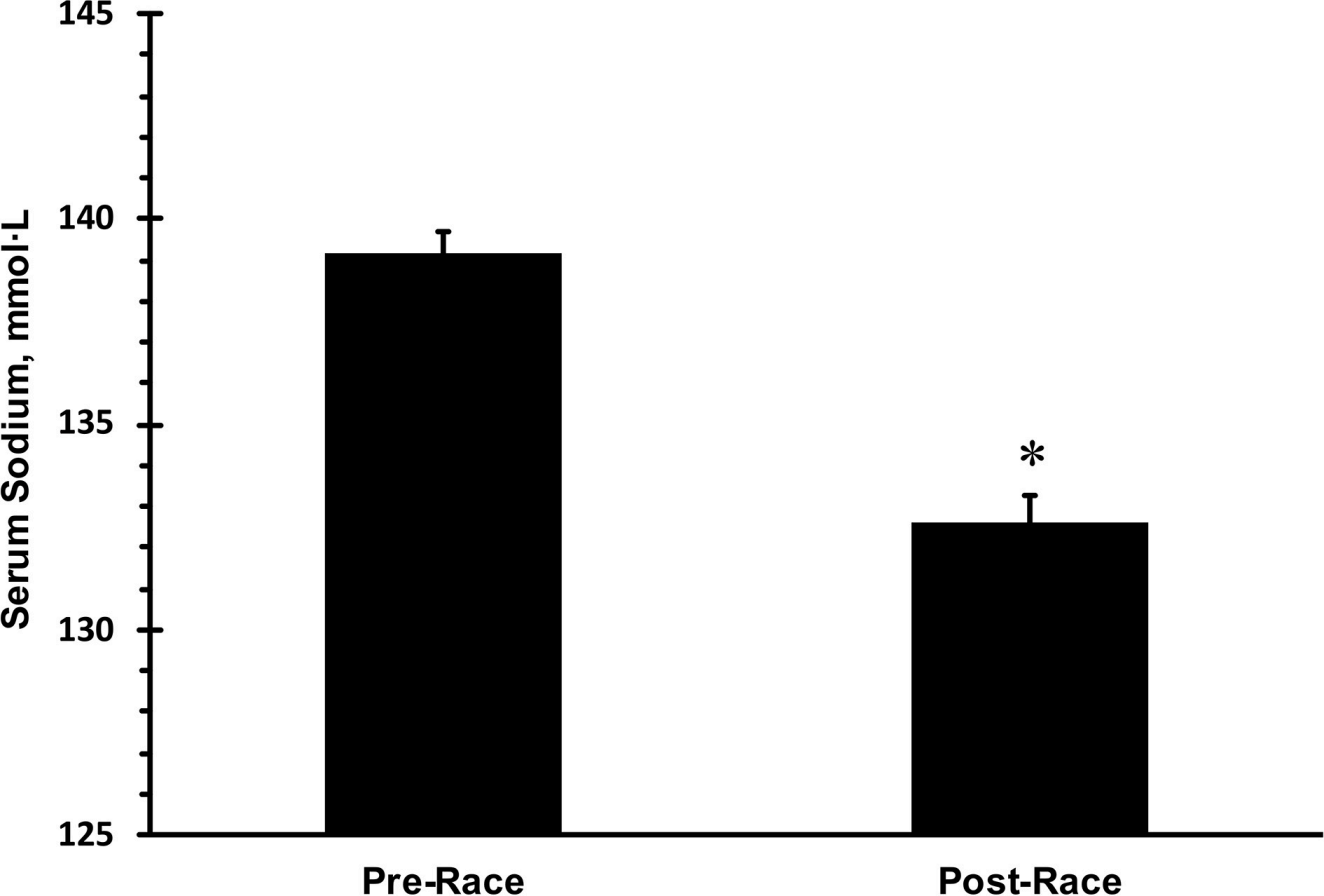
Mean pre- and post-race serum sodium in 63 finishers. ^*^statistically significant different from pre-race values. Error bars depict standard error of the mean.

**Figure 2:**
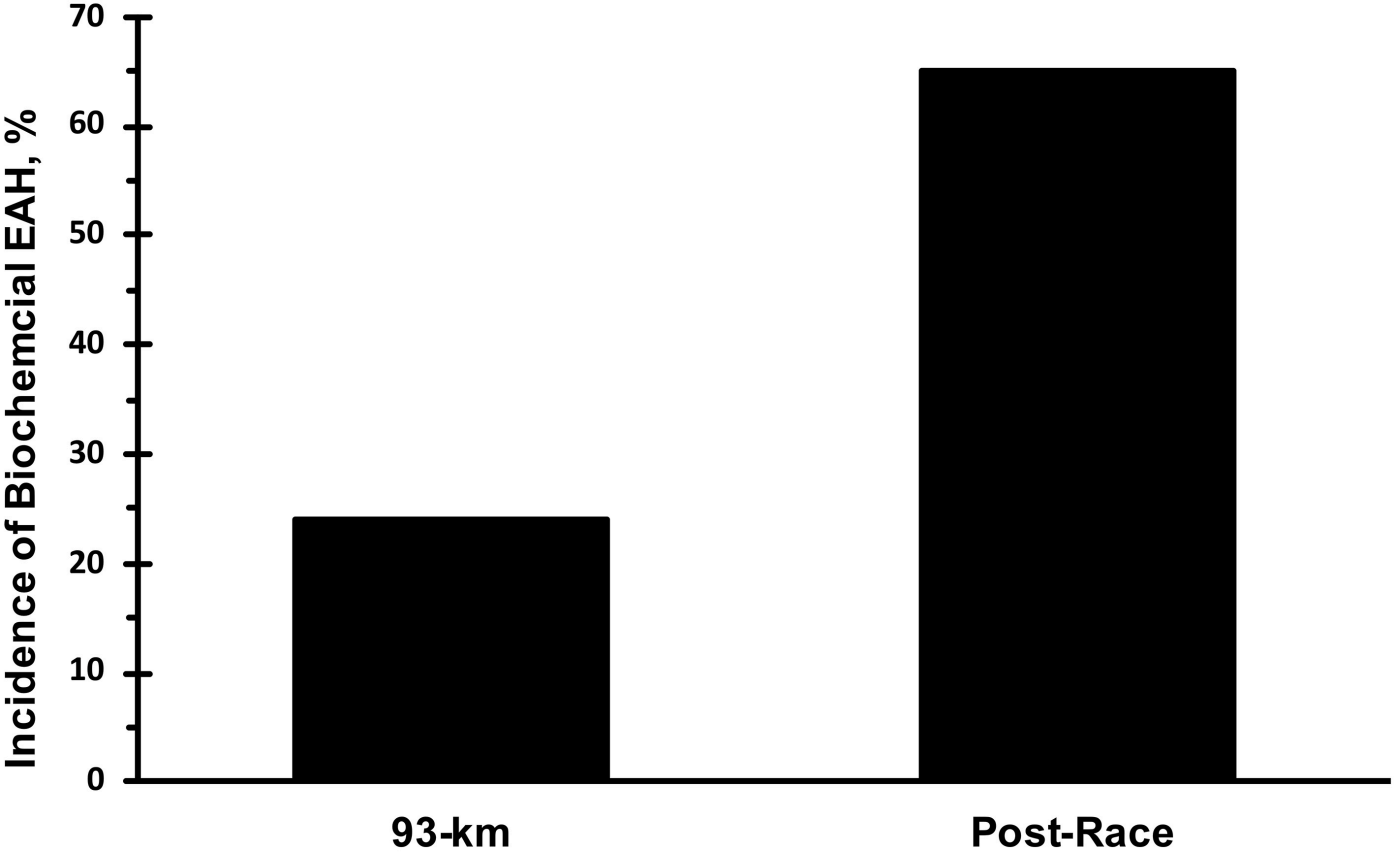
Incidence of biochemical exercise associated hyponatremia. Biochemical exercise associated hyponatremia defined as serum sodium <135 mmol·L^−1^.

**Figure 3:**
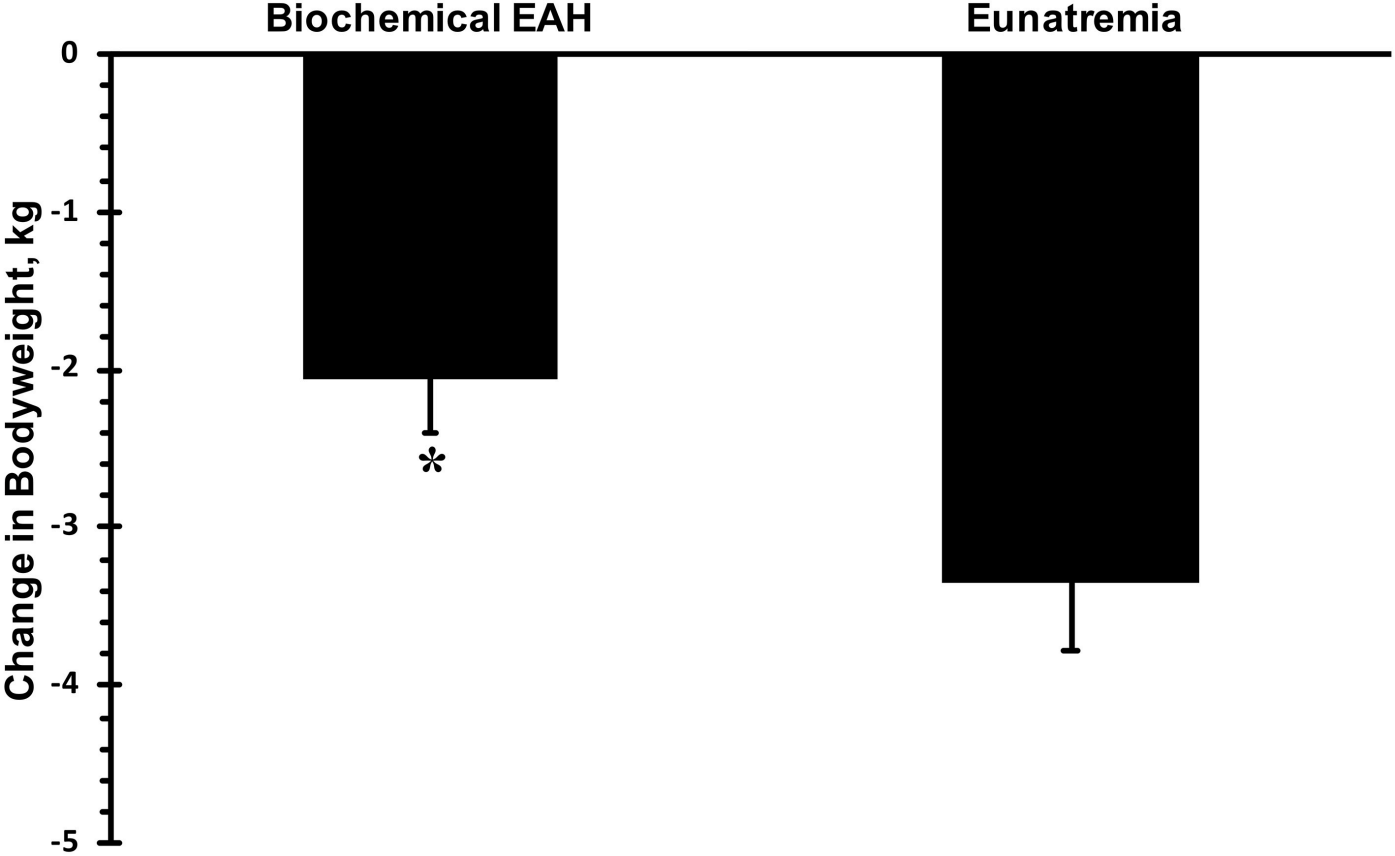
Change in bodyweight. Biochemical exercise associated hyponatremia defined as serum sodium <135 mmol·L^−1^. Eunatremia defined as serum sodium ≥135 mmol·L^−1^. ^*^significantly different from eunatremic participants.

Only data from runners who finished the race are presented. Mean race time of all subjects was 33 ± 3 h with a range of 23.5 and 36.0 h. Mean serum sodium post-race was 133 mmol·L^−1^ (Figure 1). The incidence of hyponatremia at the 93-km checkpoint (second year of data collection) and post-race (both years) were 23 and 65%, respectively (Figure 2). Of the 63 finishers recruited, nine (14%) began the race with values indicative of biochemical hyponatremia (<135 mmol·L^−1^). Eight of these 9 runners finished the race with serum sodium levels <135 mmol·L^−1^. Excluding these 8 runners from the total Incidence rate of EAH would reduce the Incidence rate to approximately 52%. Seven of 29 runners were classified as biochemically hyponatremic at the 93-km checkpoint, three of whom had sodium levels <130 mmol·L^−1^ (127, 124, 118 mmol·L^−1^). In total, 41 finishers after the race developed biochemical EAH (<135 mmol·L^−1^; *n* = 27, 43%; <130 mmol·L^−1^; *n* = 14, 22%) hyponatremia. Of the 63 finishers, none needed hospitalization or treatment for EAH related symptoms. Change in bodyweight and serum sodium from pre-race to post-race was −3.6 ± 2.7% (−2.5 ± 1.9 kg) and −6.6 ± 5.6 mmol·L^−1^, respectively. Bodyweight loss in hyponatremic competitors (−2.06 kg) was significantly smaller than bodyweight loss in eunatremic competitors (−3.36 kg) (*P* < 0.05, Figure 3).

**Figure 4:**
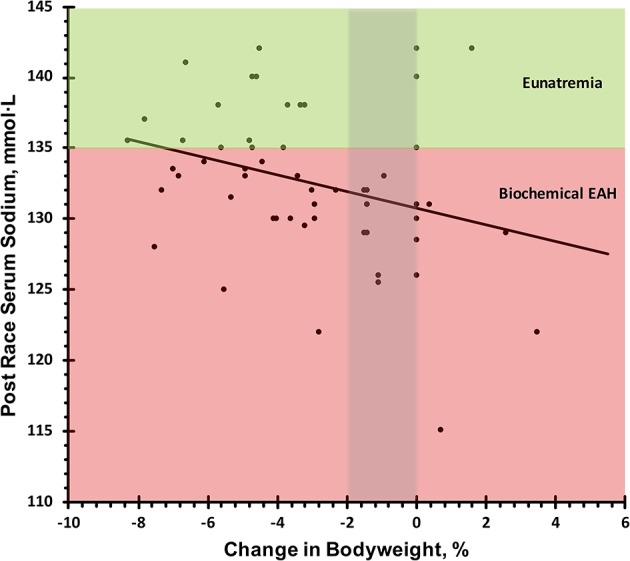
Relation of post-race serum sodium and change in bodyweight. Biochemical exercise associated hyponatremia defined as serum sodium <135 mmol·L^−1^. Eunatremia defined as serum sodium ≥135 mmol·L^−1^.

Pre-race serum sodium level was not a significant predictor of post-race serum sodium levels (β = 0.08, *R*^2^ = 0.07, *P* = 0.698); however, there was a significant negative association between change in bodyweight percentage and post-race serum sodium concentration (β = −0.79, *R*^2^ = 0.29, *P* = 0.011; Figure 4).

## Discussion

To the authors' knowledge, the observed 65% EAH Incidence rate is the highest recorded during a single event in existing literature. It is important to emphasize the extreme length, ambient conditions, and terrain undulations of the Spartathlon race as compared to other races. Although other ultramarathons feature harsher ambient conditions and steeper terrains (24, 25), the Spartathlon is considered one of the toughest ultramarathons. This most likely contributed to the rate of EAH beyond what exists in current literature.

Considering the observed Incidence rate and individual differences in sodium concentrations of sweat (7), individualized supplementation during extended ultra-marathons such as the Spartathlon may be beneficial in partially attenuating sweat sodium losses. As evident in the regression line of Figure 4, competitors who drank enough fluid to avoid significant dehydration and did not overhydrate (−2 thru 0% change in bodyweight) would still be classified as mildly hyponatremic with serum sodium levels of 131–133 mmol·L^−1^. Two studies have indicated ingestion of sodium during exercise may attenuate sweat sodium losses (16, 17). One of these, conducted by Anastasiou et al., examined differences in serum sodium as cyclists were provided beverages with differing sodium levels. Although in this study the timeframe of exercise was only 4.5 h, athletes ingesting beverages with a sodium concentration of 36.2 mmol·L^−1^ were able to maintain higher serum sodium concentrations as compared to a placebo beverage and plain water (16). However, contradictory studies have indicated ingestion of sodium during endurance events resulted in no differences in serum sodium as compared to competitors not ingesting sodium (19). Considering most sport drinks are hypotonic as compared to serum, overconsumption of these drinks can contribute to situations of dilutional hyponatremia (26).

Based on previous literature we can make a theoretical estimation of sweat sodium losses during an extreme event such as Spartathlon. Consider a runner who finished the race in 30 h with a mean sweat rate of 1 L·h^−1^ and total sweat loss of 30 L. If we assume our runner has a relatively low sweat sodium concentration of 40 mmol·L^−1^ (27), this would yield ~1.2 mol (30 L × 40 mmol·L^−1^) or 27.6 g (1.2 mol × 23 g·mol^−1^) of lost sodium throughout the event. Although this estimation does not account for all manners of sodium change, to replace this amount of sodium, athletes would need to consume 70 g of salt since 1 g of salt contains 0.39 g of sodium. This amount of sodium replacement is not feasible during a competitive race, however; smaller amounts of sodium ingestion may help partially attenuate sodium losses, especially in competitors with higher concentrations of sweat sodium (7, 16, 17). Please note that the amount of sodium loss does not include urinary or fecal losses of sodium, and these numbers represent only a moderate sweat rate and low sweat concentration. Additionally, mobilization of sodium stores from non-osmotically active stores (bone, skin, cartilage) has been proposed as a factor in the pathogenesis of EAH (28), however; this factor was not considered as this mechanism remains speculative (29).

The Spartathlon ultra-marathon is an extremely long course that has to be completed within 36 h. Additionally, the rules require the first 100-km of the race be completed within 10 h. Considering this requirement, the first section of the race is usually performed faster, and pace is significantly lowered as runners meet the 10 h distance requirement. In previous literature, increased race time was identified as a risk factor for development of EAH in shorter races (1, 26). Our data in the longer ultra-endurance Spartathlon align with previous evidence considering the large difference in EAH Incidence rate between the 93-km checkpoint and post-race measurement during which pace was significantly lowered.

Interestingly, nine of the 63 runners began the race with serum sodium levels indicative of hyponatremia, perhaps suggesting overconsumption of fluids and/or insufficient dietary sodium intake in preparation for the event. Competitive athletes may be more cognizant of their daily dietary sodium intake (30, 31) and may not ingest levels comparable to a typical Western diet. This may have also contributed to the high Incidence rate of EAH (32, 33).

Considering 65% of finishers of the Spartathlon race were classified as hyponatremic, perhaps a resistance to the symptoms of hyponatremia exists in this population. Although this study only included finishers, thereby excluding participants who possibly dropped out due to EAH symptoms, 41 total runners finished despite having serum sodium levels indicative of biochemical EAH. Furthermore, 14 finishers from the hyponatremic group had serum sodium levels <130 mmol·L^−1^. Interestingly, seven runners from the second year of data collection were classified as hyponatremic at the 93-km checkpoint, three of whom had sodium levels below 130 mmol·L^−1^ and one below 120 mmol·L^−1^. All seven of these athletes were able to finish the remaining 153-km of the race despite biochemical hyponatremia, possibly suggesting a tolerance. In a previous published study based on the same races, creatine kinase (CK) values reached 43,763 ± 6,764 IU·L^−1^ indicative of extreme body strain on these competitors (34). Despite this extreme level, none of the athletes included in this study were hospitalized or treated for any renal problems. Considering the nature of ultra-runners, they may be recurrently exposed to hyponatremic states in training and other similar events possibly serving as an explanation to this occurrence. However, it is of great importance not to underestimate the potential severity and consequences of EAH. Athletes should always remain cognizant of their competitive habits and understand how these habits affect their sodium levels. Accordingly, ultra-endurance athletes should recognize and respond appropriately to the symptoms of EAH (7).

The authors do concede several limitations. To begin with, lack of dietary intake information in this study prevents the calculation of serum sodium fluctuations due to dietary sodium intake. Furthermore, not all of the observed bodyweight changes can be attributed to fluid fluctuations. Arguments have been proposed stating that body mass loss is not a reliable indicator of dehydration due to mechanisms of loss not associated with sweat. These factors include change of mass due to substrate use, clearance of water previously bound with glycogen, and the metabolic production of water (35). These factors were not accounted for in this study, reducing the validity of body mass as a measure of dehydration. Additionally, if subjects were to drop out of the race, data could not be collected. Due to the course length, wide range of finishing times (24–36 h), and large number of checkpoints, it was not feasible to collect samples quickly from dropouts as some were transported back to Athens and others to Sparta to receive medical attention if needed. Therefore, sample size was limited to only recruited subjects able to finish the race.

Despite these limitations, the incidence of EAH of 52 and 65%, when excluding or including these individuals with pre-race hyponatremia, was the highest reported in current literature. Competitors in extreme ultra-marathons should consider the risk of EAH when planning fluid and food consumption during competition, especially in events with harsher ambient and geographical challenges similar to Spartathlon.

## Data Availability Statement

The datasets generated for this study are available on request to the corresponding author.

## Ethics Statement

The study was approved by the Harokopio University Ethics committee. All procedures performed in studies involving human participants were in accordance with the ethical standards of the institutional and/or national research committee and with the 1964 Helsinki declaration and its later amendments or comparable ethical standards.

## Dedication

The authors would like to dedicate this paper to Marcos Echegaray, who unexpectantly passed away at Cayey, Puerto Rico on May 2nd of 2019. He was training for the 2019 Comrade Marathon in South Africa.

## Author Contributions

CA, KS, ME, NY, AM, MY, and SK designed research. CA, KS, ME, NY, YT, AM, MY, FP, and SK conducted data collection and sample analysis. AS, CA, and SK analyzed the data. AS and SK wrote the paper. SK was the principal investigator and had primary responsibility for the final content. All authors read, critically revised, and approved the final manuscript.

### Conflict of Interest

AS is a scientific consultant for Gatorade Sports Science Institute, SK has served as scientific consultants for Quest Diagnostics, Standard Process, and Danone Research. SK has received grants from Danone Research. The remaining authors declare that the research was conducted in the absence of any commercial or financial relationships that could be construed as a potential conflict of interest.

## References

[1] Almond CS Shin AY Fortescue EB Mannix RC Wypij D Binstadt BA. Hyponatremia among runners in the Boston Marathon. N Engl J Med. (2005) 352:1550–6. 10.1056/NEJMoa043901 15829535

[2] Zingg MA Knechtle B Rüst CA Rosemann T Lepers R. Analysis of participation and performance in athletes by age group in ultramarathons of more than 200 km in length. Int J Gen Med. (2013) 6:209–20. 10.2147/IJGM.S43454 23589700 PMC3625029

[3] Chlíbková D Knechtle B Rosemann T Žákovská A Tomášková I. The prevalence of exercise-associated hyponatremia in 24-hour ultra-mountain bikers, 24-hour ultra-runners and multi-stage ultra-mountain bikers in the Czech Republic. J Int Soc Sports Nutr. (2014) 11:3. 10.1186/1550-2783-11-3 24512517 PMC3929155

[4] Chlíbková D Rosemann T Posch L Matoušek R Knechtle B. Pre- and post-race hydration status in hyponatremic and non-hyponatremic ultra-endurance athletes. Chin J Physiol. (2016) 59:173–83. 10.4077/CJP.2016.BAE391 27188470

[5] Chlíbková D Knechtle B Rosemann T Tomášková I Novotný J Žákovská A. Rhabdomyolysis and exercise-associated hyponatremia in ultra-bikers and ultra-runners. J Int Soc Sports Nutr. (2015) 12:29. 10.1186/s12970-015-0091-x 26113805 PMC4480906

[6] Hew-Butler T Jordaan E Stuempfle KJ Speedy DB Siegel AJ Noakes TD. Osmotic and nonosmotic regulation of arginine vasopressin during prolonged endurance exercise. J Clin Endocrinol Metab. (2008) 93:2072–8. 10.1210/jc.2007-2336 18349067 PMC2435641

[7] Montain SJ Sawka MN Wenger CB. Hyponatremia associated with exercise: risk factors and pathogenesis. Exerc Sport Sci Rev. (2001) 29:113–17. 10.1097/00003677-200107000-00005 11474958

[8] Cairns RS Hew-Butler T. Incidence of exercise-associated hyponatremia and its association with nonosmotic stimuli of arginine vasopressin in the GNW100s ultra-endurance marathon. Clin J Sport Med. (2015) 25:347–54. 10.1097/JSM.0000000000000144 25318530

[9] Lebus DK Casazza GA Hoffman MD Van Loan MD. Can changes in body mass and total body water accurately predict hyponatremia after a 161-km running race. Clin J Sport Med. (2010) 20:193–9. 10.1097/JSM.0b013e3181da53ea 20445360

[10] González-Alonso J Mora-Rodríguez R Below PR Coyle EF. Dehydration reduces cardiac output and increases systemic and cutaneous vascular resistance during exercise. J Appl Physiol. (1995) 79:1487–96. 10.1152/jappl.1995.79.5.1487 8594004

[11] Sawka MN Cheuvront SN Kenefick RW. Hypohydration and human performance: impact of environment and physiological mechanisms. Sports Med. (2015) 45(Suppl. 1):S51–60. 10.1007/s40279-015-0395-7 26553489 PMC4672008

[12] Adams JD Sekiguchi Y Suh HY Seal A Sprong C Kirkland T. Dehydration impairs cycling performance, independently of thirst: a blinded study. Med Sci Sports Exerc. (2018) 50:1697–703. 10.1249/MSS.0000000000001597 29509643

[13] Wardenaar FC Hoogervorst D Versteegen JJ van der Burg N Lambrechtse KJ Bongers CCWG. Real-time observations of food and fluid timing during a 120 km ultramarathon. Front Nutr. (2018) 5:32. 10.3389/fnut.2018.00032 29780808 PMC5946582

[14] Coyle EF. Fluid and fuel intake during exercise. J Sports Sci. (2004) 22:39–55. 10.1080/0264041031000140545 14971432

[15] Baker LB. Sweating rate and sweat sodium concentration in athletes: a review of methodology and intra/interindividual variability. Sports Med. (2017) 47(Suppl. 1):111–28. 10.1007/s40279-017-0691-5 28332116 PMC5371639

[16] Anastasiou CA Kavouras SA Arnaoutis G Gioxari A Kollia M Botoula E. Sodium replacement and plasma sodium drop during exercise in the heat when fluid intake matches fluid loss. J Athl Train. (2009) 44:117–23. 10.4085/1062-6050-44.2.117 19295955 PMC2657026

[17] Vrijens DM Rehrer NJ. Sodium-free fluid ingestion decreases plasma sodium during exercise in the heat. J Appl Physiol. (1999) 86:1847–51. 10.1152/jappl.1999.86.6.1847 10368348

[18] Koenders EE Franken CPG Cotter JD Thornton SN Rehrer NJ. Restricting dietary sodium reduces plasma sodium response to exercise in the heat. Scand J Med Sci Sports. (2017) 27:1213–20. 10.1111/sms.12748 27714955

[19] Hew-Butler TD Sharwood K Collins M Speedy D Noakes T Sodium supplementation is not required to maintain serum sodium concentrations during an Ironman triathlon. Br J Sports Med. (2006) 40:255–9. 10.1136/bjsm.2005.022418 16505084 PMC2492002

[20] Hoffman MD Stuempfle KJ. Sodium supplementation and exercise-associated hyponatremia during prolonged exercise. Med Sci Sports Exerc. (2015) 47:1781–7. 10.1249/MSS.0000000000000599 25551404

[21] Maughan RJ. Fluid and electrolyte loss and replacement in exercise. J Sports Sci. (1991) 9 Spec No:117–42. 10.1080/02640419108729870 1895359

[22] Schedl HP Maughan RJ Gisolfi CV. Intestinal absorption during rest and exercise: implications for formulating an oral rehydration solution (ORS). Proceedings of a roundtable discussion. April 21-22, 1993. Med Sci Sports Exerc. (1994) 26:267–80. 10.1249/00005768-199403000-00001 8183089

[23] McDermott BP Anderson SA Armstrong LE Casa DJ Cheuvront SN Cooper L. National Athletic Trainers' Association position statement: fluid replacement for the physically active. J Athl Train. (2017) 52:877–95. 10.4085/1062-6050-52.9.02 28985128 PMC5634236

[24] Krabak BJ Lipman GS Waite BL Rundell SD. Exercise-associated hyponatremia, hypernatremia, and hydration status in multistage ultramarathons. Wilderness Environ Med. (2017) 28:291–8. 10.1016/j.wem.2017.05.008 28781178

[25] McCubbin AJ Cox GR Broad EM. Case study: nutrition planning and intake for marathon des sables-a series of five runners. Int J Sport Nutr Exerc Metab. (2016) 26:581–7. 10.1123/ijsnem.2016-0016 27097381

[26] Hew-Butler T Rosner MH Fowkes-Godek S Dugas JP Hoffman MD Lewis DP. Statement of the 3rd international exercise-associated hyponatremia consensus development conference, Carlsbad, California, 2015. Br J Sports Med. (2015) 49:1432–46. 10.1136/bjsports-2015-095004 26227507

[27] Baker LB Barnes KA Anderson ML Passe DH Stofan JR. Normative data for regional sweat sodium concentration and whole-body sweating rate in athletes. J Sports Sci. (2016) 34:358–68. 10.1080/02640414.2015.1055291 26070030

[28] Titze J. A different view on sodium balance. Curr Opin Nephrol Hypertens. (2015) 24:14–20. 10.1097/MNH.0000000000000085 25470013

[29] Hew-Butler T Loi V Pani A Rosner MH. Exercise-associated hyponatremia: 2017 Update. Front Med. (2017) 4:21. 10.3389/fmed.2017.00021 28316971 PMC5334560

[30] Worme JD Doubt TJ Singh A Ryan CJ Moses FM Deuster PA. Dietary patterns, gastrointestinal complaints, and nutrition knowledge of recreational triathletes. Am J Clin Nutr. (1990) 51:690–7. 10.1093/ajcn/51.4.690 2321575

[31] Spendlove JK Heaney SE Gifford JA Prvan T Denyer GS O'Connor HT. Evaluation of general nutrition knowledge in elite Australian athletes. Br J Nutr. (2012) 107:1871–80. 10.1017/S0007114511005125 22018024

[32] Armstrong LE Costill DL Fink WJ Bassett D Hargreaves M Nishibata I. Effects of dietary sodium on body and muscle potassium content during heat acclimation. Eur J Appl Physiol Occup Physiol. (1985) 54:391–7. 10.1007/BF02337183 4065126

[33] Eichner ER Genetic and other determinants of Sweat Sodium. Curr Sports Med Rep. (2008) 7:S36–40. 10.1249/JSR.0b013e31817f3b35

[34] Skenderi KP Kavouras SA Anastasiou CA Yiannakouris N Matalas AL. Exertional Rhabdomyolysis during a 246-km continuous running race. Med Sci Sports Exerc. (2006) 38:1054–7. 10.1249/01.mss.0000222831.35897.5f 16775544

[35] Hoffman MD Goulet EDB Maughan RJ Considerations in the use of body mass change to estimate change in hydration status during a 161-kilometer ultramarathon running competition. Sports Med. (2017) 48:243–50. 10.1007/s40279-017-0782-3 28895063

